# Gut Microbiota Regulates Food Intake in a Rodent Model of Intermittent Limited Access to Palatable Food

**DOI:** 10.1002/eat.24339

**Published:** 2024-12-02

**Authors:** Thomas Demangeat, Léa Loison, Marion Huré, Jean‐Luc do Rego, Pierre Déchelotte, Najate Achamrah, Moïse Coëffier, David Ribet

**Affiliations:** ^1^ Univ Rouen Normandie, INSERM, Normandie Univ, ADEN UMR1073 Nutrition, Inflammation and Microbiota‐Gut‐Brain Axis, CHU Rouen, CIC‐CRB 1404 Department of Nutrition Rouen France; ^2^ Univ Rouen Normandie, INSERM US51, CNRS UAR2026, Behavioural Analysis Platform SCAC HeRacLeS Rouen France

**Keywords:** binge‐eating disorder, eating disorder, gut microbiota

## Abstract

**Objective:**

Binge‐eating disorder is characterized by recurrent episodes of consumption of large amounts of food within a short period of time, without compensatory purging behaviors. This disease is a major public health issue and is associated with numerous comorbidities, encompassing anxiety and depression. The gut microbiota has been proposed to be an important player in the onset or maintenance of eating disorders. Here, we aim to better delineate the potential role of the gut microbiota in binge‐eating disorder.

**Method:**

We used a model of intermittent limited access to palatable food where eight‐week‐old C57Bl/6 female mice had access during 2 h, every 2 days over a 10‐day period, to a high‐fat/high‐sucrose diet. Half of the animals received antibiotics to deplete their gut microbiota. Eating behavior and other behavioral parameters were compared between groups.

**Results:**

We observed an increase in food intake as well as tachyphagia during the intermittent access to high‐fat/high‐sucrose diet. We demonstrate that gut microbiota depletion further increases food intake during these episodes and promotes binge‐eating behavior. No impact on anxiety or depressive‐like behavior was observed in animals.

**Discussion:**

These results show that the gut microbiota is involved in the control of food intake during episodes of binge‐eating. This strengthens the potential role of the gut bacteria in binge‐eating disorder and the interest in therapeutic strategies aiming at modulating the patients' gut microbiota to treat this eating disorder.

**Summary**

- Binge‐Eating Disorder (BED) is a widespread yet challenging condition to treat.
- This disease is characterized by recurrent episodes of consumption of large amounts of food within a short period of time.
- The mechanisms underlying this disease are not fully understood.
- It has recently been proposed that the gut microbiota plays a significant role in the onset of this disease since gut bacteria can communicate with the central nervous system and regulate food intake.
- In this study, we evaluated the role of the gut microbiota in BED using a dedicated mouse model. Our results show that the gut microbiota regulates food intake in a model of chronic exposure to a highly palatable diet.
- Our data strengthen the hypothesis that an alteration of the gut microbiota composition is a potential susceptibility factor for BED.
- This work reinforces the interest in the development of innovative treatments based on microbiota modulation to improve the management of BED.

## 1 Introduction

Binge‐eating disorder (BED) is an eating disorder marked by recurrent episodes of consumption of large amounts of food within a short period of time (often less than 2 hours) (Wonderlich et al. 2009). These episodes are associated with an overwhelming sense of loss of control over eating. In contrast to bulimia nervosa, people with BED do not resort to vomiting or taking laxatives. In accordance with DSM‐5 criteria, BED's associated binge‐eating episodes should occur at least 1 day a week for 3 months and are frequently associated with fast eating, eating until feeling uncomfortably full, eating without feeling physically hungry, eating alone, or feeling disgusted with oneself or guilty after overeating (Diagnostic and statistical manual of mental disorders 2013). The incidence of BED has strongly increased in recent years, and more particularly since the COVID‐19 pandemic (Tavolacci, Ladner, and Dechelotte 2021). The estimated lifetime prevalence of BED is approximately 2.8% in women and 1% in men (Galmiche et al. 2019).

BED is closely linked to numerous comorbidities, encompassing anxiety, depression, functional gastrointestinal disorders, and complications associated with obesity, including metabolic disorders (Mitchell 2016). This disease exhibits striking parallels with addictive behavior and is frequently subjected to social stigma (Gearhardt et al. 2012; Ebneter and Latner 2013). As such, BED is strongly associated with distress and a reduced quality of life.

The current treatment of BED consists in regular monitoring by dietitians and nutritionists, psychotherapy, adapted physical activity, and, in some cases, the use of serotonin reuptake inhibitors (Linardon et al. 2017; Raisi et al. 2023; Arnold et al. 2002). Despite these different approaches, the success of current therapies remains limited and the relapse rate still exceed 30% at the ten‐year mark (Safer et al. 2002). This is partly due to the poor understanding of the pathophysiological mechanisms underlying BED. Factors contributing to the pathophysiology of BED encompass psychological, sociocultural, and genetic factors (Javaras et al. 2008; Treasure, Duarte, and Schmidt 2020). Additional factors, such as the gut microbiota, have recently been proposed as important players in the etiology of the disease (Guo and Xiong 2024). Indeed, the gut microbiota plays critical roles in the regulation of eating behavior, in energy metabolism as well as in the development of anxiety, depression, and functional digestive disorders (Margolis, Cryan, and Mayer 2021; Han et al. 2021). Thus, the gut microbiota may contribute to the onset and/or maintenance of BED. A gut dysbiosis has been described in BED patients, although the number of clinical studies focusing on the gut microbiota composition in this disease remains very limited (Leyrolle et al. 2021; Castellini et al. 2023). Yet, the functional consequences of this gut dysbiosis in BED pathophysiology remain unknown, and further work is required to establish causal relationships.

The use of experimental animal models is pivotal to study BED. Several animal models are described in the scientific literature that have been used to unveil the pathophysiological mechanisms of BED or to delineate the beneficial role of specific bacterial species on binge‐eating and anxiety‐like behavior (Rehn et al. 2022; Agustí et al. 2021). Most of these models are based on the exposure of rodents to high‐calorie and/or highly palatable diets for a limited duration (generally less than 2 h), in order to trigger episodes of intense food consumption mirroring those observed in BED patients. Here we used a model of intermittent limited access to palatable food to explore the potential contribution of the gut microbiota in eating behavior and comorbidities associated with BED.

## 2 Methods

### 2.1 Animals

Animal care and experimentation were approved by a regional Animal Experimentation Ethics Committee (APAFIS #38597–2022091914519369 v3) and complied with the guidelines of the European Commission for the handling of laboratory animals (Directive 2010/63/EU). All efforts were made to minimize suffering of animals.

In this study, we conducted 2 independent sets of animal experiments. In each set of experiments, we studied in parallel 4 groups, with 8 animals per group (i.e., 32 animals per experiment). We used eight‐week‐old C57Bl/6 female mice (Janvier Labs, Le‐Genest‐Saint‐Isle, France). Mice were first housed at 23°C in regular open cages with a 12 h light–dark cycle for 7 days in a ventilated cabinet. Independent cages of male mice were introduced in the same cabinet to promote the synchronization of the estrous cycles in females. Half of the female animals were treated during this first week with both antifungals and antibiotics, diluted in drinking water, to trigger gut microbiota depletion (Tirelle et al. 2020). For this, animals had first access to drinking water with Amphotericin‐B (Sigma‐Aldrich) for 3 days (0.005 mg/mL) to prevent fungal overgrowth, as a consequence of bacterial depletion (Reikvam et al. 2011). Then animals had access to drinking water containing both Amphotericin‐B (0.005 mg/mL) and Ampicillin (0.5 mg/mL, Sigma‐Aldrich), Neomycin trisulfate salt hydrate (0.5 mg/mL, Sigma‐Aldrich), Metronidazole (0.5 mg/mL, Sigma‐Aldrich), and Vancomycin hydrochloride (0.25 mg/mL, Sigma‐Aldrich) (Tirelle et al. 2020). Drinking water was renewed every day. After this first week, female mice were individually housed at 23°C with a 12 h light–dark cycle in BioDAQ cages (Research Diets Inc.), to allow for a continuous monitoring of eating behavior. Administration of antifungals and antibiotics was continued for half of the animals. Each animal was assigned to one of the following groups: a control group (CTRL group), a group periodically exposed to a high‐fat/high‐sucrose (HFHS) diet (HFHS group), a group treated with antibiotics (CTRL+ATB group), and a group both periodically exposed to a HFHS diet and treated with antibiotics (HFHS+ATB group) (Figure S1). Allocation to the different groups was performed in order to minimize body weight variations between groups.

Mice from the CTRL and CTRL+ATB groups had an *ad libitum* access to a standard diet (Teklad Global 16% Protein Rodent Diet, INOTIV) during the whole protocol. Mice from the HFHS and HFHS+ATB groups had access during 2 h (at the beginning of the dark phase), every 2 days over a 10‐day period, to a diet enriched in carbohydrates and lipids (HFHS diet, D12451i, Research Diets Inc.) (Figure S1). This diet contains 45 kcal % fat and 21 mass % sucrose. During these periods, mice from the HFHS and HFHS+ATB groups do not have access to the standard diet. Between these episodes, animals had an *ad libitum* access to the standard diet.

Body weight was monitored daily, and body composition was assessed at the end of the protocol using fast nuclear magnetic resonance (Minispec LF110, Brucker). To evaluate depressive‐like behavior, mice were subjected to a nesting test, 1 day after the last binge‐eating episode. In this test, a compressed cotton nestlet is placed in cages and a score is given to the nest constructed by mice from this cotton nestlet after 24 h. This score is based on a 5‐point nest‐rating scale as described in ref. (Deacon 2006) (a score of 1 corresponds to an almost intact nestlet and no identifiable nest and a score of 5 corresponds to a near perfect nest). To evaluate anxiety‐like behavior, mice were subjected to a light–dark compartment test 2 days after the last binge‐eating episode (Kremer et al. 2021). In this test, mice were placed in an enclosure containing two compartments, one compartment exposed to light and the other in the dark. The time spent in each compartment during the 15 first minutes of the test was recorded.

At the end of the protocol, all animals received an intraperitoneal injection of ketamine (200 mg/kg) and xylazine (20 mg/kg) and were euthanized by cervical dislocation. Mice were dissected aseptically in order to remove the cecum. Cecal contents were collected, under sterile conditions, by gently squeezing the cecum with round‐tipped forceps. Cecal contents were then stored at −80°C until analysis.

### 2.2 Quantification of Cecal Bacterial Densities

DNAs from mouse cecal contents were extracted using the QIAamp DNA Stool Mini Kit (QIAGEN), including a bead‐beating step (0.1 mm zirconia silica beads, BioSpec products), as previously described (Breton et al. 2021). Quantitative real‐time polymerase chain reaction (qPCR) was performed on these extracted DNAs to monitor the efficiency of antibiotics‐mediated gut bacterial depletion. To quantify total eubacteria, qPCR was performed using Itaq Universal SYBR Green Supermix and primers targeting conserved regions in eubacterial 16S rRNA genes (Eub‐338–F, 5’‐ACTCCTACGGGAGGCAGCAG‐3′ and Eub‐518–R, 5’‐ATTACCGCGGCTGCTGG‐3′) (Fierer et al. 2005). The Cq determined in each sample were compared with a standard curve made by diluting genomic DNA extracted from a pure culture of *E. coli* , for which cell counts were determined prior to DNA isolation.

### 2.3 Statistical Analysis

Comparison of body composition and weight variation, cumulated food intake, speed of food intake, and behavioral parameters during light–dark compartment test were performed using one‐way ANOVA with tukey's correction. Comparison of food intake and speed of food intake between groups and between episodes of access to the HFHS diet were performed using two‐way repeated ANOVA. Comparison of cumulative food intake during the course of binge‐eating episodes was performed using unpaired *t*‐test. Comparison of latency before food intake, nest scores and cecal bacterial densities were performed using kruskal–wallis test with dunn's correction. Statistical analyses were performed with GraphPad Prism 8 (GraphPad Software, San Diego, USA).

## 3 Results

We used a rodent model in which animals were exposed every 2 days over a 10‐day period to a diet enriched in carbohydrates and lipids (high‐fat/high‐sucrose (HFHS) diet; Figure S1). These short and repeated exposures to an HFHS diet aim to mimic episodes of binge‐eating. Between these episodes, animals had an *ad libitum* access to a standard diet. This model of intermittent limited access to a palatable food is similar to other protocols used to study binge‐eating behavior (Rehn et al. 2022; Valdivia et al. 2015; Blanco‐Gandía, Cantacorps, et al. 2017; Blanco‐Gandía, Ledesma, et al. 2017; Blanco‐Gandía, Minarro, et al. 2018; Blanco‐Gandía, Montagud‐Romero, et al. 2018). In order to delineate the potential role of the gut microbiota in eating behavior in this model, half of the mice were treated with antibiotics in order to deplete their gut microbiota (Tirelle et al. 2020). Antibiotics were directly added to animal's drinking water. We did not observe any decrease in water intake for mice exposed to antibiotics, nor any significant differences in water intake between the different groups treated with antibiotics. The efficiency of gut microbiota depletion was validated by quantifying the density of Eubacteria in mouse cecal contents (Figure S1).

We assigned the mice to four different groups: a control group (CTRL group), a group periodically exposed to HFHS diet (HFHS group), a group treated with antibiotics (CTRL+ATB group), and a group both periodically exposed to HFHS diet and treated with antibiotics (HFHS+ATB group) (Figure S1).

**FIGURE 1:**
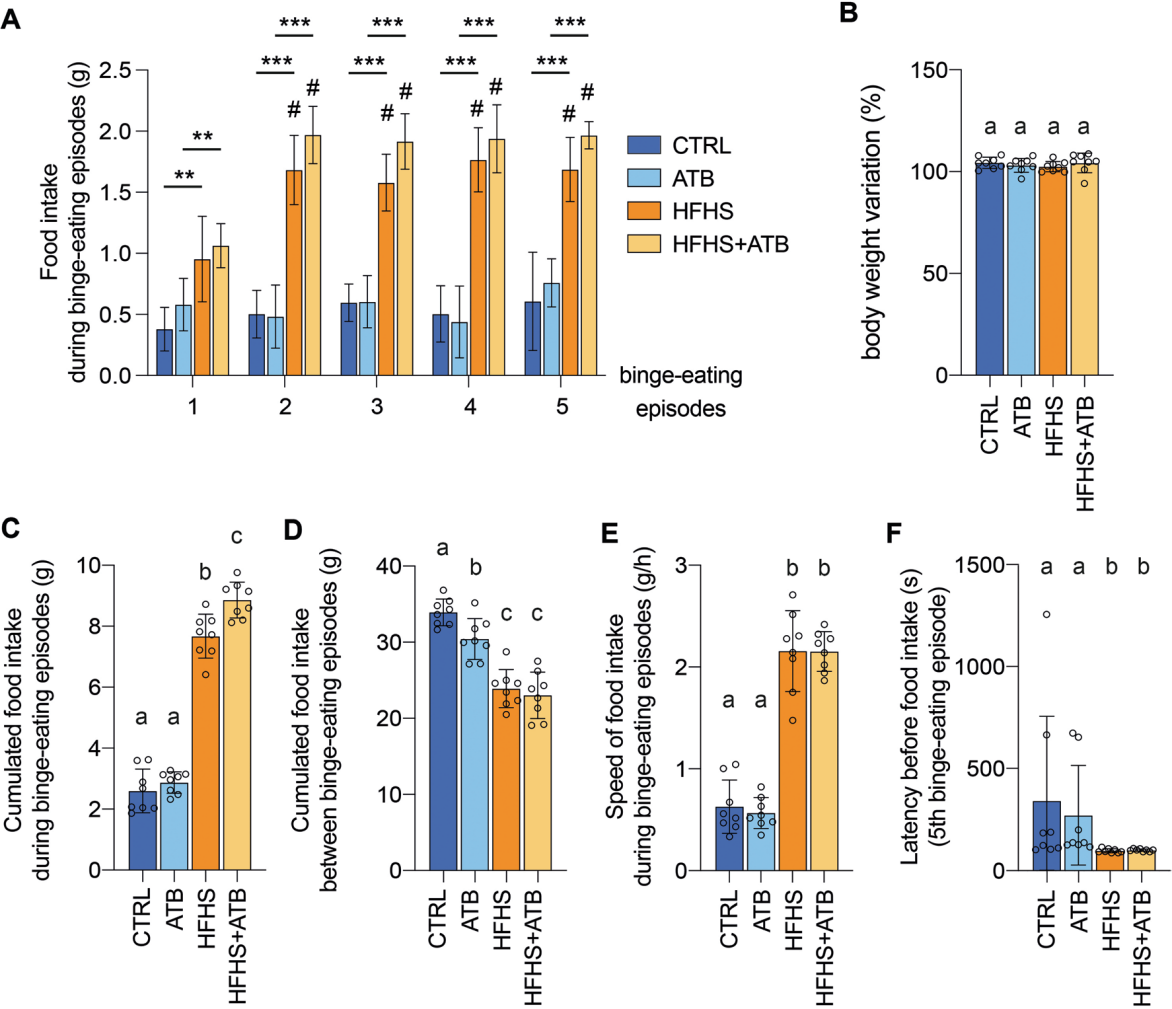
Impact of intermittent limited access to palatable food on eating behavior. (A) Food intake in each of the five binge‐eating episodes (mean ± s.d.; *n* = 8/group; 2‐way repeated ANOVA; **, *p* < 0.01; ***, *p* < 0.001; #, *p* < 0.05 vs. the first episode). (B) Body weight variation at the end of the protocol (percentage of initial body weight) (mean ± s.d.; *n* = 8/group; 1‐way ANOVA with tukey's correction). (C and D) Cumulated food intake during (C) or between (D) binge‐eating episodes (mean ± s.d.; *n* = 8/group; 1‐way ANOVA with tukey's correction). (E) Speed of food intake during binge‐eating episodes (mean ± s.d.; *n* = 8/group; 1‐way ANOVA with tukey's correction). (F) Latency between the access to standard or HFHS diet and the first event of food intake (mean ± s.d.; *n* = 8/group; kruskal–wallis test with dunn's correction). Labeled means without a common letter differ. Similar results were observed for 2 independent sets of animal experiments.

We first monitored food intake in the different groups. We observed significantly higher food intake in the HFHS and HFHS+ATB groups, compared with the CTRL and ATB groups, respectively, for each episode of access to the HFHS diet (Figure 1A). Of note, the food intake during these episodes significantly increases in HFHS and HFHS+ATB groups between the first episode and the following episodes (Figure 1A,C). We also observed that the speed of food intake is significantly higher in animals exposed to the HFHS diet compared to control animals, and increases between the first episode and the following episodes for the HFHS and HFHS+ATB groups (Figures 1E and S1). The latency between the opening of the access to food and the first food intake event is significantly decreased in animals fed with HFHS diet compared to regular diet (Figure 1F). Between these intermittent episodes of access to HFHS diet, animals from the HFHS and HFHS+ATB groups exhibit reduced food intake (Figure 1D). Accordingly, we did not observe any significant difference in body weight or body composition between CTRL and HFHS groups at the end of the protocol, despite the consumption of a diet rich in calories (Figures 1B and S1).

To assess the impact of the gut microbiota in our rodent model, we compared food intakes between the HFHS and HFHS+ATB groups. Interestingly, we observed a significant increase in the cumulated food intake of the HFHS+ATB group compared to the HFHS group during the intermittent access to HFHS diet, but not between these episodes (Figure 1C,D). This increase becomes significant with repeating exposure to HFHS diet, after the third binge‐eating episode (Figure S1). These results suggest that the depletion of the gut microbiota modifies mouse eating behavior and promotes binge‐eating behavior. This depletion does not affect the speed of food intake nor the latency before food intake (Figure 1E,F). Similar results were observed for 2 independent sets of animal experiments.

**FIGURE 2:**
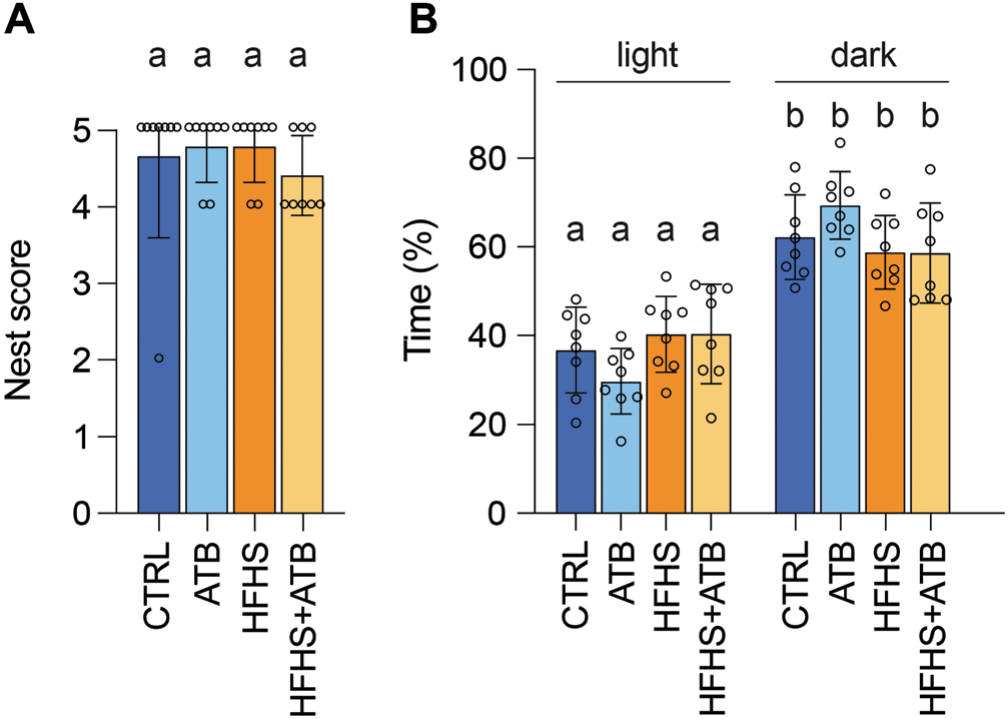
Impact of intermittent limited access to palatable food on anxiety‐ and depressive‐like disorders. (A) Nest score from the nesting test (mean ± s.d.; *n* = 8/group; kruskal–wallis test with dunn's correction). (B) Time spent in light or dark compartments during the light/dark box test (mean ± s.d.; *n* = 8/group; 1‐way ANOVA with tukey's correction). Labeled means without a common letter differ. Similar results were observed for 2 independent sets of animal experiments.

We then evaluated if the model used in this study triggers anxiety‐ or depressive‐like disorders in animals. To evaluate depressive‐like behaviors, we performed a nesting test, 1 day after the last binge‐eating episode, by providing a compressed cotton nestlet to the animals and by scoring the structure of the nest built by animals after 24 h (Deacon 2006). A poor nest score indicates depressive‐like behaviors. No significant differences in nest scores were observed between groups (Figure 2A). To evaluate anxiety, animals were placed in light/dark boxes 2 days after the last binge‐eating episode and the time spent in each compartment during the 15 first minutes of the test was recorded. The light compartment constitutes an aversive environment for rodents in this test. As such, a decrease in the time spent in this compartment is defined as an anxiety‐like behavior (Kremer et al. 2021). The time spent in the light compartment was not significantly different between groups (Figure 2B). Together, these results suggest that the model of intermittent limited access to palatable food used in this study does not trigger anxiety‐ or depressive‐like behaviors. Gut microbiota depletion has no further impact on animal behavior.

## 4 Discussion

Medical care for patients with BED is a real challenge today. Animal models are valuable tools for studying the pathophysiology of this disease and for deciphering the role of complex inter‐organ interactions, such as the microbiota‐gut‐brain axis. In order to better delineate the potential role of the gut microbiota in BED, we used here a model based on intermittent access to a highly palatable and high‐calorie‐containing food. We used mice since they usually better respond to these models than rats, and focused on females, to mirror the female predominance observed in BED (Rehn et al. 2022).

Our model mimics several important features observed in other BED animal models. This includes the escalation of food intake between the beginning and the end of the protocol, which can be reminiscent of binge‐eating, although we cannot exclude that this difference is due to neophobia associated with the introduction of a new type of food. The inclusion of an additional group of animals with an *ad libitum* (and not intermittent) access to HFHS diet could be useful in this type of study. Indeed, this “*ad libitum*” HFHS group may help to delineate whether the observed increase in food intake in the “intermittent” HFHS group is due only to the higher palatability of the diet or to the onset of *bona fide* binge‐eating behaviors (Valdivia et al. 2015). Food preference tests could also be included where animals have access to both regular and HFHS diets at the same time to monitor differences in animal preference for a particular diet. The increased rate of food intake in the HFHS and HFHS+ATB groups mimics the tachyphagia classically observed in BED patients. Interestingly, between episodes, mice decreased food consumption, suggesting that compensatory behaviors are occurring, which align with BED patients’ behavior exhibiting moderate restriction to offset excessive caloric consumption, often driven by post‐meal guilt, ultimately culminating in a loss of control over eating (Masheb and Grilo 2000). Our rodent model is distinct from an obesity model since no increase in body weight was observed. In addition, it does not recapitulate some of the classical comorbidities associated with BED such as anxiety or depression. This might be due to the short duration of our model, the lack of alternance with periods of food restriction, or the lack of stress (such as foot‐shock stress) in our experimental design, which is sometimes used in other BED models (Rehn et al. 2022). Of note, we did not observe changes in anxiety or depression in the ATB group compared to the CTRL group (Figure 2). Several studies have analyzed the impact of gut microbiota depletion triggered by antibiotics on anxiety‐like behavior. The results are actually variable since some studies reported significantly increased anxiety‐like behavior in rodents in response to ATB treatment whereas other did not report changes (Olavarría‐Ramírez et al. 2023).

We demonstrate that depletion of the gut microbiota promotes binge‐eating in our animal model. This suggests that the gut microbiota plays a role in food intake control during binge‐eating episodes. This result strongly strengthens the hypothesis that alteration of the gut microbiota, as observed in BED patients, favors loss of control in food consumption and promotes the altered eating behavior observed in BED.

Interestingly, it has been reported that antibiotic depletion of the gut microbiota in mice results in overconsumption of highly palatable food such as high‐sucrose pellets (de Wouters et al. 2021; Ousey, Boktor, and Mazmanian 2023). In these studies, the impact of gut microbiota depletion was evaluated by exposing animals only once to high‐sucrose/high‐fat diet for 2–3 h. Our work differs from these studies since we focused here on the impact of gut microbiota depletion on repeated exposures to a high‐calorie diet, using an intermittent pattern, and thus on the potential chronic development of binge‐like behaviors. Another study suggested that the gut microbiota is involved in overeating disorders, where patients present with experience of cravings for palatable food (Fan et al. 2023). In this study, a mouse model combining stress with a history of dieting was associated with a dysbiosis and an alteration of the gut metabolome, which ultimately results in an alteration of the gut‐brain axis and an hedonic overconsumption of highly palatable food (Fan et al. 2023). Of note, the model used in this study strongly differs from ours, since no restriction nor anxiety‐promoting stimuli were included in our protocol.

Altogether, our results reinforced the central role of the gut microbiota in the control of intake of highly palatable food. They further illustrate that the gut microbiota is involved in the regulation of eating behavior in models of chronic exposure to high‐calorie diet and strengthen its potential role in the onset of binge‐eating behaviors. Understanding the molecular determinants of the host‐gut bacteria cross‐talk involved in the pathophysiology of BED is now essential to design innovative therapeutic strategies aiming at manipulating patients' gut microbiota to improve the management of this eating disorder.

## Author Contributions

**Thomas Demangeat:** investigation, writing – original draft, writing – review and editing. **Léa Loison:** investigation. **Marion Huré:** investigation. **Jean‐Luc do Rego:** methodology, resources. **Pierre Déchelotte:** conceptualization, supervision. **Najate Achamrah:** conceptualization, supervision. **Moïse Coëffier:** conceptualization, funding acquisition, supervision. **David Ribet:** conceptualization, funding acquisition, supervision, visualization, writing – original draft, writing – review and editing.

## Disclosure

The authors have nothing to report.

## Supporting information


**Figure S1.** Impact of antibiotic‐mediated gut microbiota depeletion in the intermittent limited access to palatable food model.
(A) Experimental protocol. (B) Quantification of eubacteria in mouse cecal contents (mean ± s.d.; *n* = 8/group; kruskal‐wallis test with dunn’s correction). (C) Food intake cumulated over the course of binge‐eating episodes (mean ± s.d.; *n* = 8/group; unpaired *t*‐test; NS, not significant; **, *p* < 0.01). (D) Speed of food intake in each of the five binge‐eating episodes (mean ± s.d.; *n* = 8/group; 2‐way repeated ANOVA; *, *p* < 0.05; **, *p* < 0.01; ***, *p* < 0.001; #, *p* < 0.05 vs. the first episode). (E) Body composition (mean ± s.d.; *n* = 8/group; 1‐way ANOVA with tukey’s correction). Labeled means without a common letter differ. Similar results were observed for 2 independent sets of animal experiments.

## Data Availability

The authors confirm that the data supporting the findings of this study are available within the article and its Supporting Information.
