# Supplementary material for: Gut Microbiota Regulates Food Intake in a Rodent Model of Intermittent Limited Access to Palatable Food
Source: Int J Eat Disord. 2024 Dec 2;58(2):459–65. doi: 10.1002/eat.24339 (PMC11861880; doi:10.1002/eat.24339)
Supplement: Supplementary file 1 — Figure S1. Impact of antibiotic‐mediated gut microbiota depeletion in the intermittent limited access to palatable food model. (A) Experimental protocol. (B) Quantification of eubacteria in mouse cecal contents (mean ± s.d.; n = 8/group; kruskal‐wallis test with dunn’s correction). (C) Food intake cumulated over the course of binge‐eating episodes (mean ± s.d.; n = 8/group; unpaired t‐test; NS, not significant; **, p < 0.01). (D) Speed of food intake in each of the five binge‐eating episodes (mean ± s.d.; n = 8/group; 2‐way repeated ANOVA; *, p < 0.05; **, p < 0.01; ***, p < 0.001; #, p < 0.05 vs. the first episode). (E) Body composition (mean ± s.d.; n = 8/group; 1‐way ANOVA with tukey’s correction). Labeled means without a common letter differ. Similar results were observed for 2 independent sets of animal experiments. [file EAT-58-459-s001.pdf]

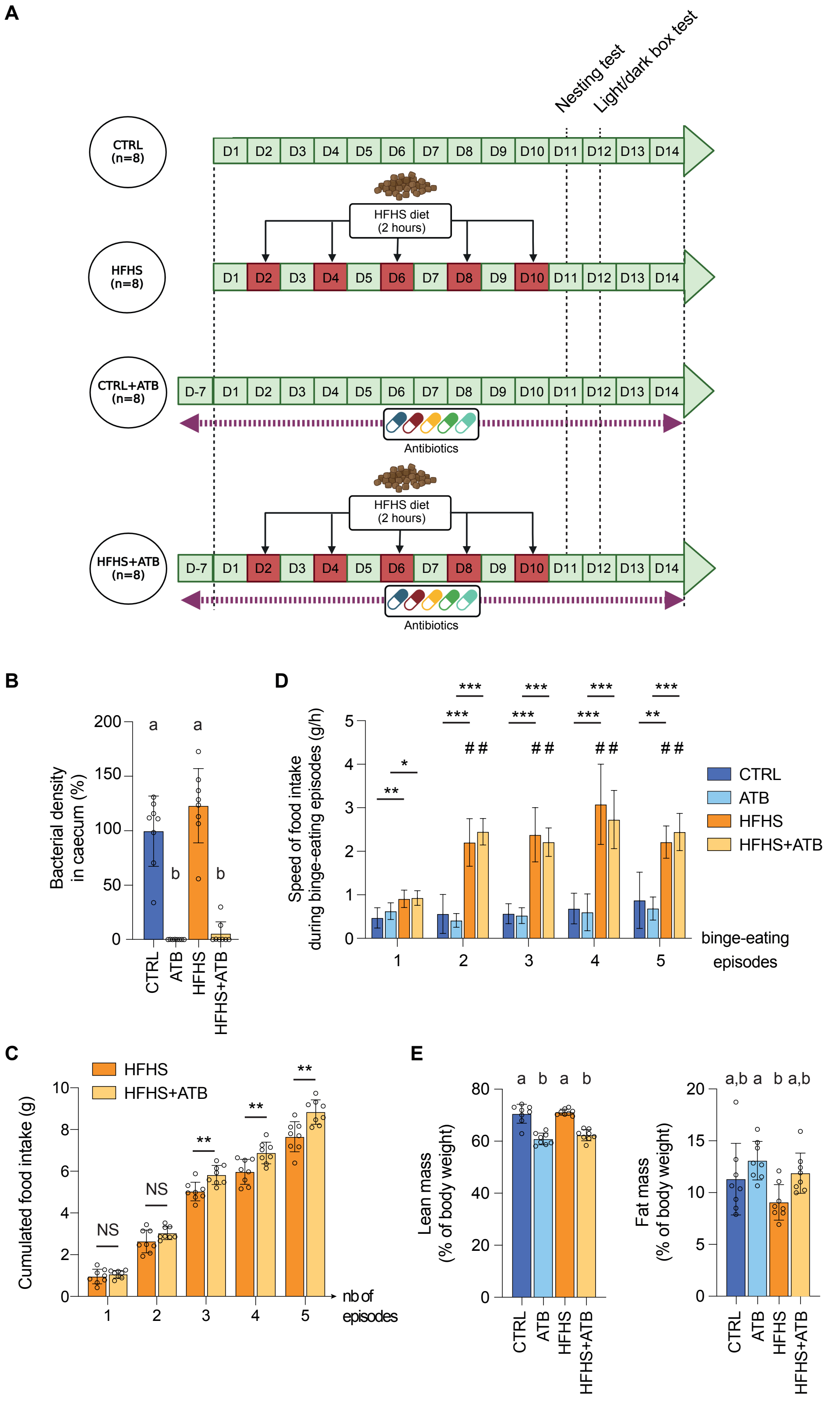

Fig. S1: Impact of antibiotic-mediated gut microbiota depletion in the intermittent limited access to palatable food model. (A) Experimental protocol. (B) Quantification of Eubacteria in mouse cecal contents (mean ± s.d.; n=8/group; kruskal-wallis test with dunn's correction). (C) Food intake cumulated over the course of binge-eating episodes (mean ± s.d.; n=8/group; unpaired t-test; NS, not significant; **, p < 0.01). (D) Speed of food intake in each of the five binge-eating episodes (mean ± s.d.; n=8/group; 2-way repeated ANOVA; *, p < 0.05; **, p < 0.01; ***, p < 0.001; #, p < 0.05 vs the first episode). (E) Body composition (mean ± s.d.; n=8/group; 1-way ANOVA with tukey's correction). Labeled means without a common letter differ. Similar results were observed for 2 independent sets of animal experiments.
